# A Review of Honeybee Venom Allergens and Allergenicity

**DOI:** 10.3390/ijms22168371

**Published:** 2021-08-04

**Authors:** Marta Burzyńska, Dorota Piasecka-Kwiatkowska

**Affiliations:** Department of Food Biochemistry and Analysis, Poznan University of Life Sciences, Mazowiecka 48 str., 60-623 Poznan, Poland; marta.burzynska@up.poznan.pl

**Keywords:** honeybee venom allergens, *Apis mellifera*, venom immunotherapy

## Abstract

Honeybee venom is a source of proteins with allergenic properties which can result in in various symptoms, ranging from local reactions through to systematic life-threatening anaphylaxis, or even death. According to the World Allergy Organization (WAO), honeybee venom allergy is one of the most common causes of anaphylaxis. Among the proteins present in honeybee venom, 12 protein fractions were registered by the World Health Organization’s Allergen Nomenclature Sub-Committee (WHO/IUIS) as allergenic. Most of them are highly immunogenic glycoproteins that cross-react with IgE and, as a consequence, may give false positive results in allergy diagnosis. Allergenic fractions are different in terms of molecular weight and biological activity. Eight of these allergenic fractions have also been identified in honey. This explains frequent adverse reactions after consuming honey in people allergic to venom and sheds new light on the causes of allergic symptoms in some individuals after honey consumption. At the same time, it also indicates the possibility of using honey as a natural source of allergen in specific immunotherapy.

## 1. Introduction

Alongside drugs and food, insect venom is one of the most frequent elicitors of anaphylaxis. In the countries of Central Europe, there are two families of *Hymenoptera* which cause allergic reactions: *Apidae*—honeybees (*Apis mellifera*) and bumblebees *(Bombus spp*.); and *Vespidae*—the common wasp (*Vespula vulgaris*) and European hornet (*Vespa crabro*). The majority of cases of anaphylactic reaction occur after a sting by honeybee, due to the higher frequency of attacks. Honeybee venom (HBV) allergy is the second most common form of allergy to *Hymenoptera* venom, and is especially prevalent in children, beekeepers and their family members, and people who are more likely to be stung [1]. While IgE specific (sIgE) to *Hymenoptera* venom are found in around 20% of the general population, sIgE antibodies to bee venom are found in 30% of honey-allergic patients [2]. Although the exact incidence of allergy to honey is unknown, it has been estimated to be less than 0.001% of the population [3]. Allergenic proteins were first identified in honey in the 1990s [4,5]. Recently, Erban et al. [6] confirmed the presence of Api m 10 (icarapine) and Api m 11b (MRJP9) in all tested honey samples (*n* = 13). Additionally, *Api* m 2 (hyaluronidase), Api m 7 (serine protease CUB) and Api m 3 (acid phosphatase) analogues were detected in eight honey samples, whereas, Api m 1 (phospholipase A2), Api m 4 (melittin) and Api m 12 (vitellogenin) were found in one sample. This information sheds new light on the causes of allergic symptoms after consumption of honey. Although pollen from nectar plants is the main protein component of honey [7], the allergenic properties of honey may also result from the presence of secretions from the salivary and hypopharyngeal glands of honeybees [8,9]. When considering the allergenic properties of honey, it should also be taken into account that honey contains pollen from wind-pollinated plants [10,11]. The average pollen content of these plants can be 6.5–7.4% [12]. To understand the causes of allergy to honey, detailed research is necessary in order to explain the mechanisms of the migration of potentially allergenic fractions into honey and the possible implications of this process. In the future, such research may not only enable the prevention of allergy to honey, but also allow for the use of honey as a new method of immunotherapy to *Hymenoptera* venom allergy (HVA) or allergies to inhaled antigens [6]. This review explores a large number of studies investigating individual allergenic molecules of the honeybee, with a focus on explaining the causes of allergenicity.

## 2. Epidemiology of Honeybee Venom Allergy

According to the World Allergy Organization (WAO), HVA is one of the most common causes of allergies in adults which can lead to anaphylaxis [13]. In Poland, the most common causes of anaphylaxis include *Hymenoptera* stings and bites (41.4%), followed by food (29.8%), and drugs and other medical products (17.4%) [14]. Sensitivity to *Hymenoptera* venom occurs in 27–40% of the general adult population and in up to 50% of children [2,15]. The risk of a systemic reaction in patients with undiagnosed allergies to the venom of black-winged insects ranges from 3.3 to 5% [2]. Large local injection site reactions occur in up to 26% of the general population and are defined as swellings larger than 10 cm in diameter lasting 24–48 h [16,17]. A total of 15–20% of general population tests positive for IgE (sIgE), but most people do not experience dangerous symptoms after a sting. Allergic reactions are more often observed in adults. Anaphylaxis occurs in 0.3 to 7.5% (or 0.85–5%, according to other sources) of sting cases [18]. In beekeepers, the statistics are different. In this group, excessive local reactions occur in 38% of sting cases (11.8% in Poland), and generalised ones in 14–43% cases (8.9% in Poland) [19,20]. According to the available data, in Poland the number of people who die every year due to hypersensitisation caused by venom ranges from several to a dozen or so every year. Based on the data collected worldwide to date, the death rate related to stings is 0.09–0.13 per million people/year [13].

## 3. Mechanism of *Hymenoptera* Venom Allergy (HVA)

Allergy is a hypersensitivity reaction of the immune system and, according Gel and Combs, classification can be categorised into four subtypes (Table 1) [21]. 

While there are a variety of mechanisms of the *Hymenoptera* venom allergy (HVA), the most common mechanism is the type I hypersensitivity reaction mediated by a venom allergen-specific IgE bound to the high-affinity FcRI receptor on mast cells and basophils. On allergen cross-linking of these receptors, mediators (e.g., histamine) are released [22]. Symptoms following a honeybee sting usually appear within 30 min and are often accompanied by skin reactions, such as itching, redness, hives and angioedema, while systemic reactions, including anaphylaxis, are relatively rare in the general population [23]. However, some people with high concentrations of sIgE do not show clinical manifestations of allergy, and some patients with very low or even undetectable levels of sIgE may experience life-threatening systemic reactions. There is evidence that the complement system may play an important role in the manifestation of the clinical symptoms of allergy to bee venom [24]. There are also other possible mechanisms of allergy to *Hymenoptera* venom in the form of type III (immune complex reaction) and type IV (delayed-type reaction) hypersensitivity reactions [25]. A type III reaction can lead to various syndromes, depending on the antibodies produced. The production of IgG antibodies directed against venom antigens may result in a serum sickness-like syndrome and glomerulonephritis caused by the deposition of immune complexes containing IgG and venom antigens [20]. Even in 20% of people who are allergic, the hypersensitivity reaction may be biphasic, with the second phase developing 1–72 h after the sting [26]. Responses to stings can also take the form of nonimmune (i.e., toxic) reactions. 

In addition, most allergens of *Hymenoptera* venom are glycoproteins, in which the carbohydrate residue, an alpha-1,3 fucose bond to the N-glycan core (CCD—cross-reacting carbohydrate determinant), is highly immunogenic, cross-reacting with IgE. This, as a consequence, may give false-positive results of allergy diagnosis [27,28,29]. This mechanism is primarily found in patients with double sensitisation to the venom of yellow jackets or honeybees [30,31] and/or with polysensitisation to pollen allergens [32]. Glycoproteins are very common in pollen, food and *Hymenoptera* venoms. Studies on the presence of anti-CCD IgE in the sera of allergy sufferers indicate that the presence of these antibodies does not cause visible clinical symptoms [19,23,27,28]. For example, patients allergic to grass pollen, whose serum IgE reacts with *Phl* p4, one of the allergens found in timothy grass (*Phleum pratense*), also reacted to Api m1, even though they were not sensitised to bee venom [33]. Glycosylated proteins with CCD have a high frequency of IgE reactivity but low allergenicity, which is why sensitised individuals develop IgE antibodies against the antigenic CCD. Therefore, the positive diagnosis in patients sensitive to *Hymenoptera* venom can be also due to the presence of CCD-sIgE in their serum [34].

## 4. Symptoms of Bee Sting Allergy

A normal local reaction (NR) is the most common symptom of a bee sting. It is characterized by the appearance of slight swelling, erythema and pain after the sting. The symptoms may last for any time from a few minutes up to several hours. The reaction may be more severe if the sting affects the mucosa (e.g., the mouth) or an area rich in loose connective tissue (e.g., the eyelids, fingertips or lips). In addition, there may be complications in the form of sting site infection, accompanied by a fever and enlargement of the surrounding lymph nodes. Some allergy sufferers may develop a large local reaction (LLR), which is caused by the late phase of the allergic reaction. This involves a swelling more than 10 cm in diameter and lasting more than 24 h, which intensifies 6–12 h after the sting and reaches its largest size after 24–48 h. Swelling may be accompanied by malaise, chills, fever or headache, or an inflammatory reaction along the lymphatic vessels. The aforementioned changes resolve slowly and gradually over several days. Large local reactions may be life-threatening if the sting site is in the area of the upper respiratory tract, but the risk of a generalised reaction is not significantly increased in the case of subsequent stings [35]. Another classification of symptoms is the breakdown of a generalised allergic reaction according to the classical four-grade scale developed by Mueller [27] (Table 2).

Another type of symptoms is a generalised toxic (non-immune) reaction, which appears in patients who have been stung by many insects at the same time and is based on the description of their symptoms, including: rhabdomyolysis; damage to the heart muscle; liver or kidney dysfunction; haemolytic anaemia; haemorrhagic diathesis; and disseminated intravascular coagulation syndrome. The fifth classification of symptoms includes very rare, atypical reactions (e.g., serum sickness, vasculitis or Guillain-Barre syndrome). Cardiac arrhythmias and acute coronary syndrome may occur during anaphylaxis. There is also a specific form of anaphylaxis referred to as allergic myocardial infarction (Kounis syndrome), caused by the narrowing of coronary arteries under the influence of mediators released from mast cells in the vascular wall. The first symptoms of an anaphylactic reaction usually appear a few minutes after the sting and disappear completely within the following few hours [25].

In anaphylactic shock after the injection of the *Hymenoptera* venom, common gastric symptoms such as nausea and vomiting are observed, with tingling in the arms and legs and restlessness. Severe anaphylaxis affects the respiratory and cardiovascular systems. The characteristic symptoms are bronchospasm and shortness of breath, tachycardia, hypotension, sweating and loss of consciousness. Urinary and faecal incontinence occurs with severe circulatory dysregulation, and the most severe systemic reactions lead to cardiac and respiratory arrest. When cardiovascular or respiratory symptoms appear after initial skin manifestations such as urticaria, the clinical diagnosis of HVA is easier [23].

## 5. Venom Immunotherapy

Causal treatment in the form of allergen immunotherapy (AIT), called venom immunotherapy (VIT) in the case of the *Hymenoptera* venom, is an effective therapy for the allergic symptoms of stings in people allergic to a proven IgE-dependent mechanism of the reaction. Immunotherapy with the venom of wasps and bees (VIT) is the only method used to radically reduce the risk of a systemic reaction to the stings of these species of insects (*Hymenoptera* venom allergy systemic allergic reactions—HVA-SYS) [36].

Qualification for VIT is based on clinical criteria and diagnostic tests confirming the presence of sIgE against insect venoms. Systemic reaction is understood as a generalised IgE-dependent reaction of the body to an insect sting and is classified on a four-grade scale of reaction severity developed by Mueller (1966) [27] (Table 2). Insect venom allergen immunotherapy is recommended for patients with life-threatening HVA symptoms—HVA-SYS III-IV, for people with a high degree of exposure to stings (bee breeders) or people with comorbidities increasing the risk of a severe reaction [29,33].

VIT involves the repeated administration of increasing doses of the allergen (aqueous solutions or extracts of venom adsorbed on aluminium hydroxide) in order to achieve tolerance to the allergen. For HVA, only injection immunotherapy is used. The initial phase of the treatment (VIT induction), usually starts with a dose of 1 µg of venom. The dose of the allergen is then gradually increased in order to reach the standard value, (i.e., 100 μg of venom). Upon reaching the maintenance dose, the patient demonstrates clinical tolerance (short-term efficacy). For long-term effectiveness, it is necessary to continue therapy for the next 3–5 years [31].

The objective of immunotherapy is to direct the immune response from humoral to cellular immunity. The intention of specific immunotherapy is to reduce the synthesis of IgE antibodies by B lymphocytes through to exposure to escalating doses of an allergen administered under the control of an allergist. During treatment, the concentration of the allergen is increasing. This increase encourages the production of IL-12 by Langerhans cells and macrophages. Following this, IL-12 inhibits the production of Th2-type cytokines (IL-4, IL-5, and IL-13) and stimulates the synthesis of Th1-type cytokines (including IFN-γ). As a result of these processes, the production of IgE is inhibited. The presence of IL-10, which strongly inhibits the production of specific IgE and increases the level of specific IgG4, also plays an important role in the mechanism of immunotherapy. This occurs because IL-10 inhibits the excitation of mast cells and eosinophils and is responsible for the induction of T-cell anergy [37,38,39]. Specific immunotherapy involves the risk of several side effects, both local and systemic in nature. Systemic complications are found in 8–20% of patients undergoing therapy. They appear 3–6 times more often during immunotherapy with bee venom than with wasp venom, and more often during the initial phase than during the maintenance phase [35,39]. In a study by Kolaczek et al. [33], the side effects of immunotherapy occurred in 64 out of 180 study participants (43.8%). Early and late side effects of VIT were more common in the maintenance phase than in the induction phase (26.7%/17.8%), while systemic adverse reactions were more common in the induction phase of immunotherapy. Most of these adverse reactions were early reactions (up to 30 min after the administration of a dose of immunotherapy) and they were observed in 4 patients who had been desensitised with wasp venom (2.7%) and in 6 patients who had been desensitised with bee venom (17.6%). In only one case was the administration of adrenaline required. Early and late side effects in both the induction phase and the maintenance phase were more common in patients who were allergic to bee venom [34]. Antigen-specific immunotherapy is currently the only causal treatment of allergic diseases in bee venom allergy [40]. The applied treatment reduces the allergen sensitivity in an allergic person, resulting in the elimination or reduction of the severity of disease symptoms. The beneficial effect of the treatment is maintained for many years after it is completed [35]. Boyle et al. [41] argue that insect venom immunotherapy is an effective method of preventing further allergic reactions to insect stings, which may improve the quality of life for people suffering from bee venom allergies. However it is important to note that treatment with bee venom extract carries a low but nonetheless significant risk of systemic side effects which may affect up to 40% of patients [42]. In this context, the economic analysis showed that VIT is cost-effective only in individuals who are stung frequently (e.g., beekeepers) or in cases where an improvement in the quality of life for the patient is considered [43]. This prompts a search for new immunotherapeutic strategies that will be safer, more convenient, widely available, free from side effects and will provide high treatment effectiveness.

## 6. The Major Honeybee Allergens

The World Health Organization’s Allergen Nomenclature Sub-Committee (WHO/IUIS) has established a method to classify known allergens. This system is based on the name of the genus, the species from which a given allergen has been isolated and the number used to mark the sequentially reported allergens [44]. A total of 12 allergenic fractions from the honeybee (*Apis mellifera*) are known and registered, and they can be found in the official database of allergens of the WHO/IUIS Allergen Nomenclature Sub-Committee [45]. As many as 11 of these allergens come from bee venom (Api m 1–10, Api m 12), while two allergenic isoforms are derived from bee secretions from the royal jelly-producing glands (*Api m 11a* (0101) and *Api m 11b* (0201)).

Bee venom glands produce venom, which is a complex mixture of compounds that includes proteins, peptides, amino acids, phospholipids, sugars, biogenic amines, volatile compounds, pheromones, and a water content close to 80% [46,47,48]. The composition of HBV proteins is highly complex, including at least 113 identified proteins and peptides [49]. The complexity is increased further by N-glycosylation sites and the heterogeneity of proteins (e.g., Api m 1 [50,51] or Api m 6 [52,53]). The allergenic components of bee venom are enzymes and glycoproteins with a molecular weight ranging from 3 to 200 kDa. Honeybee venom also contains numerous small-molecule substances, including pheromones and peptides showing a local toxicity. In terms of their biological role, allergens identified in the venom are divided into major and weaker allergens, assuming that a percentage of over 50% of allergic persons will have positive specific IgE antibodies for a particular component of the venom as a criterion for distinguishing between them [54]. Pheromones are compounds used for communication (e.g., they convey to other bees information about imminent danger and the need to defend themselves). On the other hand, the main constituent of honeybee venom consists of enzymatic proteins, such as hyaluronidase, acid phosphatase and phospholipase A2, as well as peptides and non-enzymatic proteins including melittin, secapin, MCD-peptide, apamine (which is a neurotoxin), procamine and minimin. From the point of view of allergic reactions, the following are distinguished: phospholipase A2 (Api m 1)—an enzyme with a cytotoxic effect; hyaluronidase (Api m 2); acid phosphatase (Api m 3); and melittin (Api m 4) with haemolytic properties that can stimulate and adversely affect heart rate. Other components of honeybee venom include vasoactive amines: histamine, which causes pain and lowers blood pressure; dopamine and catecholamines with locally high cytotoxic potential, whose extent may increase in the case of multiple stings [55,56,57,58,59]. The overview of honeybee (*Apis Meliffera*) venom allergens, including the number of potential glycosylation sites, are summarised in Table 3 whereas Table 4 presents the rates of sensitisation to recombinant HVA in HBV patients.

**Figure 1:**
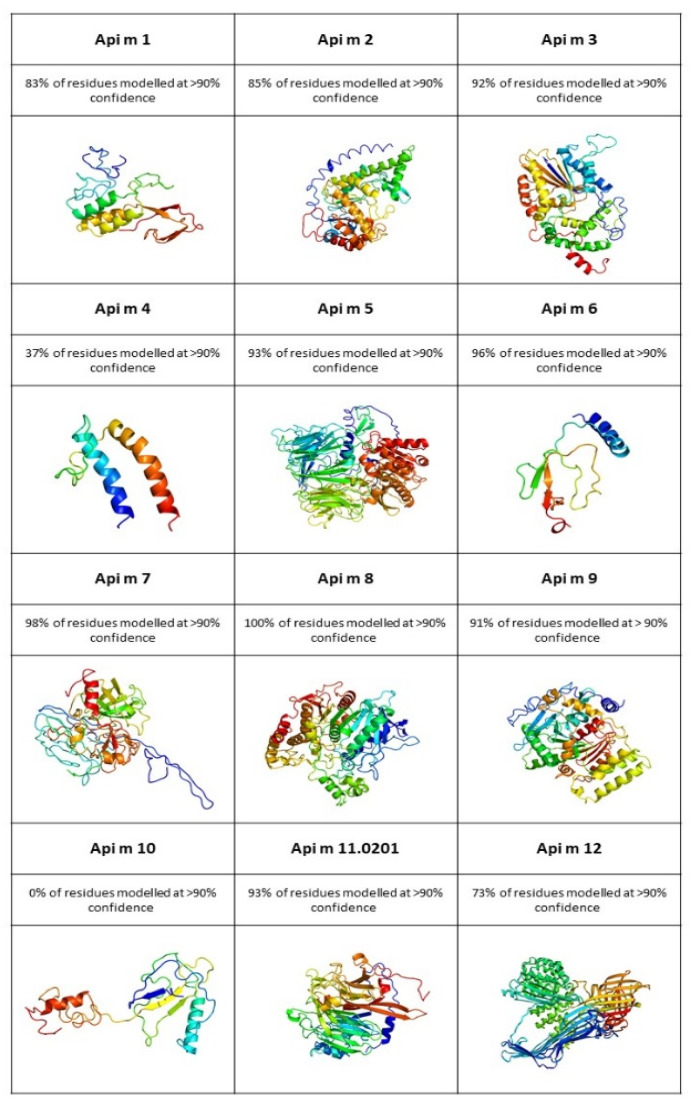
Structural features of selected *Hymenoptera* venom allergens. Three-dimensional structures of all HBV, 100.0%–90.0% confidence by the single highest scoring template, Image rainbow-colouring from N to (C-terminus). The structures were generated by structural modelling (PHYRE2 server [60,61]).

Figure 1 presents structures generated on PHYRE2 server 3D structures models of HBV allergens [60,61]. All models were prepared with a high confidence value (>90%), making the models confident. The percentage of residues modelled is informative regarding the equivalence to identical template residues in the generated alignment. For extremally high accuracy models, the percentage should be above 30–40%, but even at very low percentage (<15%) they can be very useful as long the confidence is high [62]. The amino acid sequences of allergens were loaded from the UniProt/Swiss-Prot protein knowledgebase [63] and intensive mode was chosen for modelling.

Api m 1 is a phospholipase A2 (EC: 3.1.1.4) (short name: bvPLA2), which is an enzyme protein with a molecular weight of about 16 kDa, which is one of the main allergens of bee venom. It accounts for 10–12% of the dry weight of bee venom and is the most active known phospholipase [78]. Phospholipase A2 consists of a single chain of 128 amino acid residues and contains attached carbohydrate residues. The secretion of this protein into the venom follows a seasonal pattern. This variation is synchronised with the melittin variation (i.e., their production increases at the same pace) [79]. The allergenic effect of Api m 1 is related to the activity of an enzyme that is involved in the degradation of cell membrane lipids. The secreted phospholipases A2 are enzyme proteins with a molecular weight of 14 to 18 kDa, which contain about 6–8 disulphide bridges that stabilise the proper tertiary structure of the enzyme [80]. Phospholipase A2 belongs to the acyl hydrolases, catalysing the calcium-dependent hydrolysis of the 2-acyl groups in 3-sn-phosphoglycerides [81]. During this reaction, fatty acids (e.g., arachidonic acid) and lysophospholipid are formed, causing degradation of the structure of cell membranes. These enzymes are responsible for the formation of intracellular eicosanoid signalling molecules, such as arachidonic acid metabolites, which can act as secondary messengers in the central nervous system. Secreted phospholipases are also involved in the process of endo- and exocytosis and reorganisation of the cytoskeleton. Under the influence of the enzymatic activity of phospholipases, hydrolysis of membrane phospholipids occurs and the membrane loses its potential and becomes permeable to Ca^2+^ ions. This increase in the concentration of Ca^2+^ ions in the cytoplasm causes disturbances in mitochondrial function. In addition, cytoskeleton-degrading calcium-dependent proteases are activated. It also results in the activation of calcium-dependent cytosolic phospholipases hydrolising intracellular membranes and, consequently, disintegrating the nerve cell [82]. IgE antibodies specific to Api m 1 are present in the sera of 97% of individuals allergic to bee venom [68]. The presence of IgE reactivity to commercially available rApi m 1 was detected in 72.2% of patients with HBV allergy (*n* = 144) with the use of ImmunoCap [67]. Table 3 presents the rates of sensitisation to recombinant Api m 1 in HBV patients noted by different authors. Api m 1 is the first allergenic fraction that was identified in the tested honey samples [6].

Api m 2 is hyaluronidase (EC: 3.2.1.35) (short name: Hya), which is, according to the WHO/IUIS classification, a protein with a molecular weight of 44 kDa, showing hyaluronidase activity and containing 382 amino acids. Although Api m 2 only accounts for 1–2% of the dry weight of honeybee venom, it is considered to be its second major allergen. Hyaluronidases are the enzymes which are responsible for the degradation of hyaluronic acid, thus increasing the permeability of connective tissue and reducing the viscosity of body fluids, which facilitates the spread of toxins and injected fluids. One of the three groups of hyaluronidases are endo β-N-acetylhexosaminidases (EC: 3.2.1.35). These are the enzymes found in mammals, as well as in the venoms of snakes and *Hymenoptera* species [83]. They hydrolyse β-1,4-glycosidic bonds in hyaluronic acid, yielding the main decomposition products of hyaluronic acid: tetra and hexasaccharides [84]. Aside from their hydrolytic activity, the enzymes of this group can also transglycosylate, which results in the formation of di-, tri-, -octa- and nonasaccharide units during the degradation of hyaluronic acid [85]. Hyaluronidase found in the venom of *Hymenoptera* (bees, wasps, hornets) may cause allergic reactions, including anaphylactic shock. About half of the population allergic to bee venom have hyaluronidase-specific IgE antibodies [78]. Due to the similarity of the structures of bee and wasp hyaluronidase, this allergen is the main cause of cross-reactions with specific IgE antibodies directed against the venom of both insects [86]. However, in the opinion of some authors, the cross-reactivity of IgE antibodies between bee and wasp venoms is not due to the similar structure of their hyaluronidases but instead due to the reactions with cross-reacting carbohydrate determinants (CCDs) which are secreted by these insects. This thesis calls into doubt the role of hyaluronidase as the second most allergy-provoking component of bee venom after phospholipase A2 [87]. In people allergic to *Hymenoptera* venom, the incidence of anti-CCD IgE is generally more than 20% [88]. IgE reactivity to commercially available recombinant Api m 2 was demonstrated in 50.0% of patients with a HBV allergy (Table 3). Moreover, Erban et al. [6] detected the Api m 2 allergen in eight of the analysed types of honey.

Api m 3 is a venom acid phosphatase Acph-1, which is a protein with a weight of 43kDa belonging to the histidine acid phosphatase family (EC: 3.1.3.2), also known as phosphomonoesterase, which exhibits optimum activity in an acidic environment (pH 4.4–4.8). It was first identified in the venom of the honeybee in the 1960s by Benton [89]. The allergen comprises 373 amino acids and contains 3 potential N-glycosylation sites and 2 disulphide bonds [71]. The isoelectric point of the protein is 5.64. Acid phosphatase detected in honeybee venom is an acidic protein resistant to higher temperatures [57]. Similarly to hyaluronidase (Api m 2), it is present in bee venom in a small amount, constituting only 1–2% of the venom [78]. Antibodies to acid phosphatase are detected in 60% of allergy sufferers (Table 3). Acid phosphatase can stimulate sensitised basophils to release histamine, but the mechanism of its action is not fully explained [90]. As such, an analysis were carried out that revealed the presence of analogues to Api m 3 (acid phosphatase) across all tested types of honey (*n* = 13) [6].

Api m 4 is a melittin (short name: MEL/MLT) [1.C.18.1.1] which is a cytotoxic 26 amino acid peptide with a low molecular weight of 3kDa of a hydrophobic nature (except for the hydrophilic C-terminus). It is found in two forms, due to cis-trans isomerisation at 56-Leu-Pro-57, in which the trans conformation is the major form [91]. Melittin is the main toxin found in bee venom with strong haemolytic and antimicrobial activities [92]. It is the main component of the weight of bee venom, accounting for about half of its dry weight (40–60%) [93]. Api m 4 has allergenic properties much lower than those of phospholipase A2 or hyaluronidase. Melittin acts on the lipid layer of cell membranes and has strong lytic properties. This peptide acts synergistically with phospholipase A2. Melittin attaches to the lipid bilayer and disrupts its structure, resulting in the susceptibility of membrane phospholipids to the enzymatic action of phospholipase A2. As a consequence, these phospholipids cause cell lysis (they are responsible for erythrocyte haemolysis). Api m 4 is also the main pain-inducing compound. It causes pain by activating primary nociceptor cells directly and indirectly, due to its ability to activate plasma membrane phospholipase A2 and its pore-forming activity [94]. Bee venom is characterised by bacteriostatic and bactericidal properties, precisely due to the presence of melittin. Api m 4 shows strong cytotoxic and antiviral properties, as demonstrated in vitro studies on HIV-1. This peptide was found to directly inhibit the activity of the main structural genes of this retrovirus: gag and po [95]. Melittin is responsible for the cardiotoxicity of bee venom, causing both dysfunctions of heart contractions and morphological changes (e.g., balloon degeneration) [96]. This peptide can cause bradycardia, arrhythmias, and atrioventricular blocks [97]. Melittin also interferes with the transport of certain substances through the membranes of renal tubular cells [98]. It is also responsible for the local inflammation and pain sensations around the site of a bee sting [99]. The secretion of this protein into the venom follows a seasonal pattern. This variation is synchronised with the phospholipase A2 variation (i.e., their production increases at the same rate) [79]. The sera of 25–50% of patients allergic to bee venom contain the antibodies against Api m 4 [90]. Api m 4 was detected in only one of the analysed types of honey [6].

Api m 5 is a protein with a molecular weight of 100 kDa with dipeptidyl peptidase IV activity. Due to the structural similarity of this enzyme to the enzyme found in wasp venom (*V. vulgaris*)—*Ves* v 3 is the second most common cause (after hyaluronidase) of allergenic cross-reactions between these insects [90]. Dipeptidyl peptidase IV is responsible for converting promelittin to melittin in the bee’s venous tract and modulating the chemotactic activity of immune cells following insect stings [77,95]. Api m 5, also known as allergen C, is likely to be another major allergen recognised by sIgE in most HBV allergic patients [72]. However, it does not cross-react with other major bee venom allergens, including Api m 1, Api m 2, Api m 3, and Api m 4 [100].

Api m 6 constitutes only 1–2% of the dry weight of venom and is considered to be one of the weakest allergens of bee venom because the presence of specific IgE antibodies against this allergen was found in the blood serum in less than 40% of people allergic to venom [54,74,91]. It is a basic protein with a mass of 8kDa, a low molecular weight, and an isoelectric point of 9.7 occurring in 4 isoforms (*Api m 6.01* to *6.04*) with the activity of a serine protease inhibitor [53,73]. Individual isoforms of this allergen have similar molecular weights and primary structures, with a characteristic central sequence consisting of 67 amino acids. They differ in up to 6 amino acids found at their amino and carboxyl terminals. There are 10 cysteine residues in the polypeptide chain, forming 5 disulphide bonds and stabilising the loop characteristic of canonical inhibitors, which is an epitope recognised by sIgE. In bee venom, all Api m 6 isoforms occur in equimolar amounts and play a similar role in allergic reactions [53,73].

Api m 7 is a 39 kDa molecular weight protein with protease activity. The chain consists of 245 amino acids forming two domains, serine protease like (SPL) and CUB domain (CUB-protease) [101]. The structure of Api m 7 suggests that the CUB domain is involved in interactions with natural substrates, while the SPL domain is likely to activate zymogens [102]. Api m 7 shows high IgE binding activity. As many as 80% of sera of people allergic to bee venom confirmed immunoreactivity with the 39 kDa fraction [101]. Additionally, an analysis was conducted that showed the presence of analogues to Api m 7 (CUB serine protease) across all tested kinds of honey [6].

Api m 8 is a carboxylesterase-6 (EC: 3.1.1.1) that belongs to the type-B carboxylesterase/lipase family with a molecular weight of 70 kDa. The protein consists of 536 amino acids, is glycosylated in 4 positions, and also has two disulphide bridges. Api m 8 is a negligible part of bee venom, constituting less than 1% of it, and its function is not yet fully explained [90]. On the other hand, 46% of serum samples from 28 individuals allergic to bee venom contained IgE, which reacted with *rApi m 8.0101* [74].

Api m 9 is a venom serine carboxypeptidase (EC: 3.4.16.5), belonging to the peptidase S10 family. The protein consists of 449 amino acids and has 5 N-glycosylation sites [103]. There is no information regarding immunogenic properties of Api m 9.

Api m 10 is the glycoprotein with a molecular weight of 50–55 kDa composed of 223 amino acids that was first identified by Peiren et al. [50]. The name proposed for Api m 10 is icarapine, an artificial term linking “Icarus” from Greek mythology and the genus name “*Apis*” and indicating its unstable nature and rapid degradation [104].

About four out of five patients diagnosed with bee venom allergy show a specific IgE binding reaction to icarapine. Therefore, it is considered to be the major bee venom allergen, although its content in venom is marginal (less than 1% DM). In addition, it is an allergen with activity independent of the cross-reacting CCD bicarbonate residues, which makes it a potentially crucial element to be used in the diagnosis of bee venom allergy [104]. Two best-known isoforms of Api m 10 are variant 1 and 2 [52], both of which show independent IgE reactivity of cross-reactive carbohydrates (CCDs) [52,75]. A further seven additional Api m10 allergen isoforms present in the venom gland have recently been identified [105]. The immunoreactive properties of Api m 10 were first assessed on a larger scale in 2011 by Blank et al. [75]. They used a recombinant allergen devoid of cross-reactive carbohydrate determinants (CCD) in the ELISA test, and the results showed that 53% (27/51) of patients allergic to HBV and YJV and 47% (8/17) of patients with HBV showed sIgE up to Api m 10. Studies published by Köhler et al. [67] showed allergy to Api m 10 (≥0.35 kUA/l) in 62% (89/144) of patients allergic to HBV. In 2015, Api m 10 became commercially available (ImmunoCAP ™) for routine diagnosis. Shortly thereafter, Frick et al. [106] retrospectively analysed Api m 10 sensitisation in a cohort of patients prior to HBV VIT and found Api m 10 sIgE (≥0.35 kUA/l) in 72% (83/115) of patients. Erban et al. [6], in turn, have confirmed the presence of Api m 10 in all tested honey samples.

Api m 11 occurs in two isoforms: major royal jelly protein (MRJP) 8 (*Api m 11.0101a*), with a molecular weight of 65 kDa, and MRJP9 (*Api m 11.0201a*), with a molecular mass of 60 kDa. Royal jelly proteins (MRJP) are a family of highly homologous proteins, so far identified only in the genus *Apis*, which constitutes ~90% of all royal jelly (RJ) proteins [107]. Royal jelly is a substance produced by the secretory glands of worker bees and used to feed the larvae, which consists of 400 amino acids and contains six N-glycosylation sites. MRJP9 consists of 403 amino acids with three N-linked glycosylation sites [107]. MRJP9 has been identified independently in three different organs of the honeybee: antennae, in the mandibular glands [108], and the venom sac [50]. Human IgE antibodies recognizing MRJP1 are present in the sera of patients allergic to RJ, but also in the sera of people allergic to bee venom (38% of respondents) and in 52% of patients with respiratory and/or digestive allergies [109]. The authors concluded that IgE antibodies may arise in response to commonly inhaled allergens that cross-react with RJ proteins. The unequivocal identification of MRJP9 in bee venom by Peiren et al. [50] led to the hypothesis that the presence of this protein in bee venom may be one of the possible factors responsible for the development of allergies to RJ, at least in some sIgE patients. Consequently, some individuals allergic to RJ (without prior contact with RJ) may have developed an allergy through contact with the MRJP9 that is present in bee venom. The sequence similarity between MRJP1 and MRJP9 (and other royal jelly proteins) is high (74%), so some immunological epitopes are likely common to the MRJP family of proteins. Antibodies against one MRJP have been reported to cross-react with the remaining RJ proteins [110]. According to Albert and Klaudiny [111], it is necessary to test the significance of the presented immunological and allergic effects. Nevertheless, for a full clarification of this issue, it is recommended that patients who are overreactive to bee stings monitor their symptoms carefully after consuming products containing RJ (e.g., honey). Prophylactic testing for MRJP-responsive antibodies in these patients can be effective in preventing life-threatening allergic reactions [112]. In a study by Erban et al., all of the honey samples which were tasted contained Api m 11 both isoforms MRJPs, 8 and 9 [6].

Api m 12 is a vitellogenin, with a molecular weight of 200 kDa. In most animals, both invertebrates (including honeybees) and vertebrates, vitellogenin is involved in the formation of the yolk during oogenesis [113]. Blank et al. [77] identified two vitellogenins in the venom of honeybees (Api m 12) and *Vespula vulgaris* (*Ves* v 6) using protein sequencing based on tandem mass spectrometry. The recombinant proteins of both allergens are glycosylated but CCD-free, since no reaction with rabbit anti-HRP (horseradish peroxidase) serum specific for α-1,3 core N-glycan fucosylation was detected. This feature excludes the occurrence of cross-reactivity, pointing to Api m 12 and *Ves* v 6 as interesting alternatives for improving the diagnosis of HBV and YJV allergies. In honey, Api m 12 is present very rarely, and was detected in only one of thirteen analysed samples [6].

## 7. Conclusions

Due to the complexity of the composition of bee venom and the mechanisms of its distribution, the venom released by the bee may be one of many combinations that differ slightly in their content. This fact complicates the diagnosis of bee venom allergy, which is based on still-imperfect skin and serological tests. Additionally, eight venom allergens can also be found in honey. This creates the connection between honey and honeybee venom allergy and can explain their coexistence.

Honey allergy symptoms leading to anaphylaxis are relatively rare, but an increasing trend in the consumption of natural and unprocessed foods may increase the incidence of honey allergy. From this point of view, honey ought to be considered as the source of dangerous HVA. However, in honey, the amount of honeybee venom allergenic fractions is rather low, and honey can thus potentially be helpful in venom immunotherapy as the natural source of allergenic fractions. Furthermore, honey is also the source of biologically active plant pollen, of which some can also be allergenic which may limit such an application. Despite the multitude of studies on the allergenicity of bee venom and honey, there are still many issues that need to be clarified. Certainly, detailed studies explaining the reasons for the presence or absence of individual allergenic fractions of bee venom in honey are necessary. In the future, this would make it possible to develop more effective immunotherapies based on honey for people who are allergic to bee venom, without side effects.

## Figures and Tables

**Table 1:** Classical Gel Combs classification of hypersensitive reactions.

Type of Hypersensitivity	Mediated by	Effector Mechanism
I	IgE	mast cells degranulation and release of histamine and other inflammatory mediators—immediate reaction
II	IgG, IgM	leading to the complement system activation and cell damage or lysis—cytotoxic reaction
III	IgG, IgM sometimes IgA	build-up immune complexes resulting in complement system activation, which leads to polymorphonuclear leukocytes (PMNs) chemotaxis and eventually causing tissue damage—complex reactions
IV	T-cells	T-cells or macrophages are activated as result of cytokine release leading to tissue damage—delayed-type reaction

**Table 2:** Severity degree of allergy to *Hymenoptera* venom according to Mueller scale [22].

Degree	Symptoms after a Sting
0	extensive local reaction (swelling > 10 cm lasting > 24 h)
I	hives, itching
II	any symptom listed above and at least two of the following: Quincke’s oedema (as a single symptom qualifies as degree II), chest tightness, nausea, vomiting, diarrhoea, abdominal colic, dizziness
III	any of the abovementioned symptoms and at least two of the following: dyspnoea (as a single symptom qualifies as degree III), wheezing (as a single symptom qualifies as degree III), wheezing (as a single symptom it qualifies as degree III) and dysphagia, weakness, a sense of being in a life-threatening situation, anxiety
IV	any symptoms listed above and at least two of the following: drop in blood pressure, fainting, loss of consciousness, urinary and stool incontinence, cyanosis

**Table 3:** Characteristics of allergenic fractions of honeybee (*Apis Mellifera*) according to WHO/IUIS Allergen Nomenclature Sub-Committee [45], characterisation and position of N-glycosylation according to the UniProt/Swiss-Prot protein knowledgebase [63].

Allergen	Tissues	Biochemical Name	MW [kDa]	Chain	Route of Allergen Exposure	Dry Weight [%]	Position of N-glycosylation
Api m 1	Venom gland	Phospholipase A2	16	167	Injection	10–12	46
Api m 2	Venom gland	Hyaluronidase	44	382	Injection	1–3	115; 263
Api m 3	Venom gland	Venom acid phosphatase Acph-1	43	388	Injection	1–2	182; 228; 366
Api m 4	Venom gland	Melittin	3	70	Injection	~50	-
Api m 5	Venom duct	Dipeptidylpeptidase IV	100	775	Injection	>1	68; 239; 473; 505; 578; 631; 689; 694
Api m 6	Venom gland	*Api* m 6.03/ *Api* m 6.04	8	92	Injection	1–2	-
Api m 7	Venom duct	CUB serine protease	39	405	Injection	>1	113; 209; 229
Api m 8	Venom duct	Carboxylesterase	70	557	Injection	>1	145; 374; 478; 528; 542
Api m 9	Venom duct	Serine carboxypeptidase	60	467	Injection	>1	130; 169; 304; 322; 344
Api m 10	Venom duct	Icarapin variant 2, carbohydrate-rich protein	50–55	223	Injection	>1	126;142;168;193
Api m 11 *Api m 11a* (0101)	Hypopharyngeal glands	Major royal jelly protein (MRJP)—deglycosylated forms MRJP 8	60	415	Injection/ food	-	
*Api m 11b* (0201)		MRJP 9	65	423			
Api m 12	Venom gland	Vitellogenin	200		Injection	-	296; 1067

**Table 4:** Rates of sensitisation to honeybee venom recombinant allergens in *Hymenoptera* venom allergic patients.

Allergen	Sensitisation Frequency (%)	No. of Patients	References
Api m 1	79	34	[64]
	57	175	[65]
	78	100	[66]
	72	144	[67]
	97	100	[68]
	68	16	[69]
	126/83	54/66	[70]
Api m 2	48	144	[67]
Api m 3	50	144	[67]
	38	40	[71]
Api m 4	27	82	[64]
	42	40	[66]
	23	144	[67]
Api m 5	60	35	[72]
	58	144	[67]
Api m 6	26	31	[73]
Api m 8	28	46	[74]
Api m 10	49	68	[75]
	62	144	[67]
*Api m 11a* (0101) *Api m 11b* (0201)	15/34	47	[76]
Api m 12	44	45	[77]

## Data Availability

Not applicable.

## Abbreviations

WAO	The World Allergy Organization
WHO/IUIS	The World Health Organization’s Allergen Nomenclature Committee
sIgE	Specific Immunoglobulin E
MRJP	Major Royal Jelly Protein
HVA	*Hymenoptera* Venom Allergy
CCD	Cross-reacting Carbohydrate Determinant
LLR	Large Local Reaction
NR	Normal Local Reaction
AIT	Allergen Immunotherapy
VIT	Venom Immunotherapy
HVA-SYS	*Hymenoptera* Venom Allergy Systemic
HBV	Honeybee Venom
